# The Clinical Value of Explainable Deep Learning for Diagnosing Fungal Keratitis Using *in vivo* Confocal Microscopy Images

**DOI:** 10.3389/fmed.2021.797616

**Published:** 2021-12-14

**Authors:** Fan Xu, Li Jiang, Wenjing He, Guangyi Huang, Yiyi Hong, Fen Tang, Jian Lv, Yunru Lin, Yikun Qin, Rushi Lan, Xipeng Pan, Siming Zeng, Min Li, Qi Chen, Ningning Tang

**Affiliations:** ^1^Guangxi Health Commission Key Laboratory of Ophthalmology and Related Systemic Diseases Artificial Intelligence Screening Technology & Research Center of Ophthalmology, Guangxi Academy of Medical Sciences & Department of Ophthalmology, The People's Hospital of Guangxi Zhuang Autonomous Region, Nanning, China; ^2^China-ASEAN Information Harbor Co., Ltd., Nanning, China; ^3^Guangxi Key Laboratory of Image and Graphic Intelligent Processing, Guilin University of Electronic Technology, Guilin, China; ^4^Department of Radiology, Guangdong Provincial People's Hospital, Guangdong Academy of Medical Sciences, Guangzhou, China

**Keywords:** fungal keratitis, *in vivo* confocal microscopy, deep learning, artificial intelligence, explainability

## Abstract

**Background:** Artificial intelligence (AI) has great potential to detect fungal keratitis using *in vivo* confocal microscopy images, but its clinical value remains unclarified. A major limitation of its clinical utility is the lack of explainability and interpretability.

**Methods:** An explainable AI (XAI) system based on Gradient-weighted Class Activation Mapping (Grad-CAM) and Guided Grad-CAM was established. In this randomized controlled trial, nine ophthalmologists (three expert ophthalmologists, three competent ophthalmologists, and three novice ophthalmologists) read images in each of the conditions: unassisted, AI-assisted, or XAI-assisted. In unassisted condition, only the original IVCM images were shown to the readers. AI assistance comprised a histogram of model prediction probability. For XAI assistance, explanatory maps were additionally shown. The accuracy, sensitivity, and specificity were calculated against an adjudicated reference standard. Moreover, the time spent was measured.

**Results:** Both forms of algorithmic assistance increased the accuracy and sensitivity of competent and novice ophthalmologists significantly without reducing specificity. The improvement was more pronounced in XAI-assisted condition than that in AI-assisted condition. Time spent with XAI assistance was not significantly different from that without assistance.

**Conclusion:** AI has shown great promise in improving the accuracy of ophthalmologists. The inexperienced readers are more likely to benefit from the XAI system. With better interpretability and explainability, XAI-assistance can boost ophthalmologist performance beyond what is achievable by the reader alone or with black-box AI assistance.

## Introduction

Fungal keratitis (FK) is one of the most common causes of cornea-derived blindness (1) but the diagnosis and treatment of this disease remain difficult (2, 3). Corneal smears and cultures are the gold standard for diagnosing FK (4). However, culture routinely takes several days before the results are available. *In vivo* confocal microscopy (IVCM) is a useful method for the diagnosis of FK, which allows non-invasive and *in vivo* detection of even subtle changes in the living cornea (5, 6). IVCM shows a variety of cellular changes in cornea suffering from FK (7) and foremost among these is the presence of hyphae, which is considered the specific manifestation of filamentous fungi infection (8, 9). Correct and prompt monitoring of the fungal hyphae in IVCM images contributes to make a diagnosis of FK as early as possible and optimize the appropriate management of patients (10). Manual analysis of the IVCM images, however, is extremely labor-intensive, time consuming, and is heavily dependent on observer experience (11).

Recent advances in deep learning (DL) promise to improve diagnostic accuracy, thereby improving the quality of patient care. In our previous study, DL-based models were successfully developed to detect FK in IVCM images with high accuracy (12, 13). However, the impact of these methods in clinical settings remains unclarified. A major shortcoming in the application of the DL technology to artificial intelligence (AI)-assisted medical care is the inability to interpret the model decision. From a clinical perspective, interpretability and explainability is essential for gaining clinicians' trust, for establishing a robust decision-making system, and to help overcome regulatory issues. However, DL models conceal the rationale for their conclusions, and therefore lack an understandable medical explanation to support their decision-making process, which seriously restricts its clinical application.

Explainable AI (XAI) is an important title and an active research direction in the field of medical AI research (14). A straightforward and effective strategy is to generate meaningful heatmaps that visualizes which pixel regions of an input image are important for the decision made by the DL model. Toward this objective, many approaches have been proposed for the explainable analysis of medical images, including dimension reduction, feature importance, attention mechanism, knowledge distillation and surrogate representations (15–22). Among these methods, Class Activation Mapping (CAM) offers a valid approach by performing global average pooling on the convolutional feature maps and mapping back the weights of the classification output to the convolutional layer (23). However, CAM requires altering the network architecture and re-training the network, which limits its application in different kinds of networks. Gradient-weighted Class Activation Mapping (Grad-CAM) is a generalization of CAM (24). Grad-CAM computes the neuron importance weights by performing global average pooling on gradients via backpropagation, enables the creation of class-discriminative visual explanations from much more complex networks. Inspired by this, researches have been proposed to build XAI modules to determine the most predictive lesion areas in computed tomography images (25). However, XAI approaches have not been validated in the analysis of IVCM images.

In this study, we developed an XAI-based system to diagnose FK using IVCM images and provided visual explanations based on Grad-CAM and Guided Grad-CAM methods to highlight the relevance for the decision of individual pixel regions in the input image. We compared the performance of ophthalmologists with the assistance of the black-box AI model and the explainable system, and investigated the potential of the XAI-assisted strategy to help ophthalmologists identify the causative agent of corneal infection.

## Materials and Methods

### Study Design and Datasets

A total of 1,089 IVCM images collected from Guangxi Zhuang Autonomous Region People's Hospital were finally included in the testing set in this randomized controlled trial. The images were obtained from eyes diagnosed with fungal keratitis or bacterial keratitis in the Department of Ophthalmology between June 2020 and July 2021. All the infections were confirmed by culture or biopsy. Of the 1,089 images, 522 were collected from 17 eyes with fungal keratitis and were identified as hyphae-positive, and 567 were collected from 18 eyes with bacterial keratitis and were identified as hyphae-negative. The images were acquired following a standard operating procedure with IVCM (HRT III/RCM Heidelberg Engineering, Germany). All images were screened and the poor-quality images were excluded. This study was conducted in compliance with the Declaration of Helsinki and approved by the ethics committee of The People's Hospital of Guangxi Zhuang Autonomous Region. Informed consent was waived because of the retrospective nature of the study and anonymized usage of images.

All images were independently adjudicated by three corneal specialists with over 15 years of experience. Each image was classified as hyphae-positive or hyphae-negative. A reference standard for each image was generated when consistent diagnostic outcomes were achieved by the three specialists. None of the adjudicators were included as readers in this study.

### Classification Model and Visual Explanation

The development of the DL-based diagnostic model used in this study is described in detail in our previous study (12). Briefly, the model was trained using the Residual Learning network-101 convolutional neural network architecture. The training set consisted of 2,088 IVCM images that had reference standard labels agreed by a panel of corneal experts. The image was input with the dimensions of 384 × 384. The classification model was trained to output the prediction probability of negative and positive classes.

**Figure 1:**
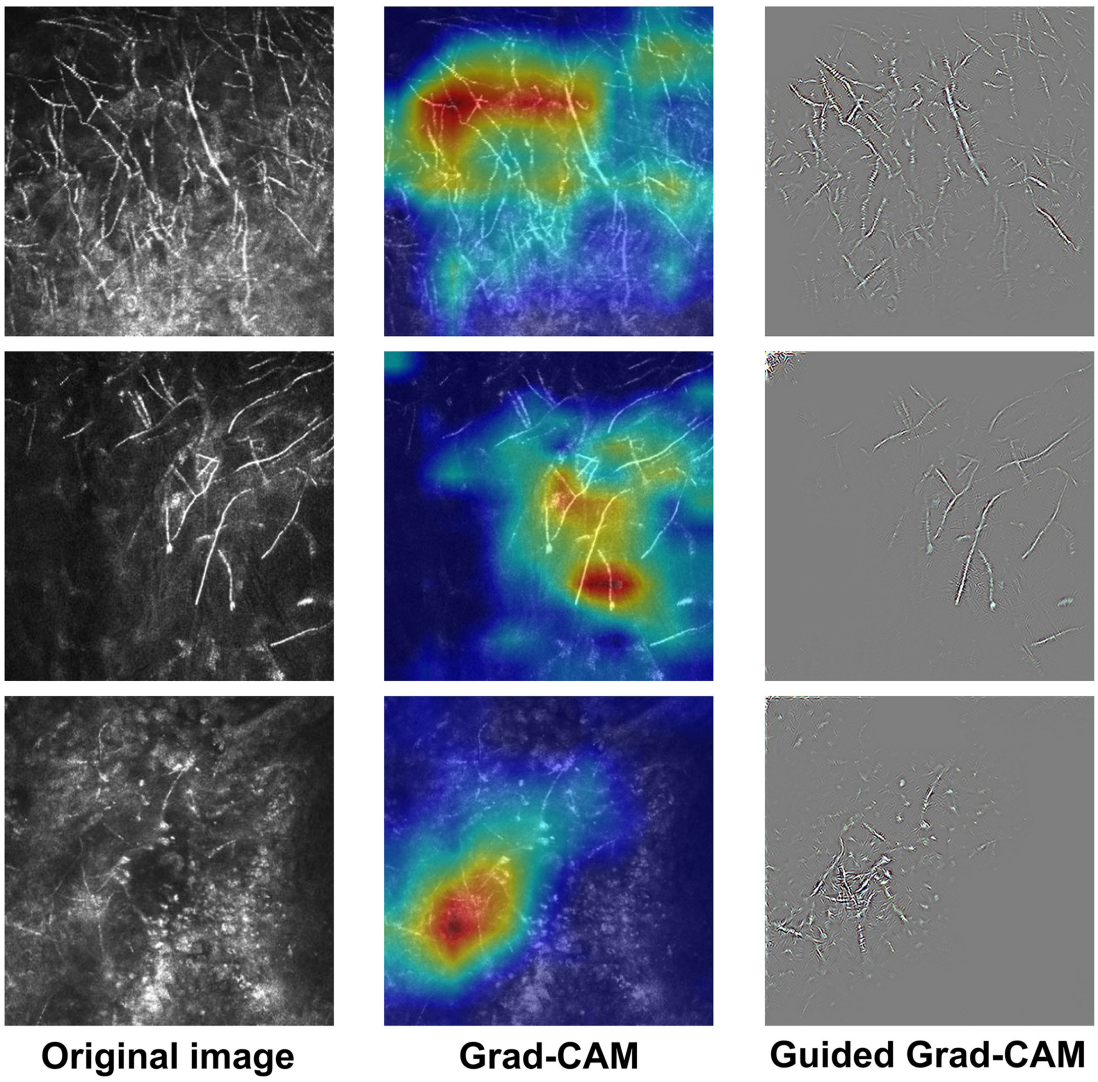
Examples of IVCM images and the corresponding explanatory maps. Original images **(left)**, Grad-CAM **(middle)**, and Guided Grad-CAM **(right)**.

We used Grad-CAM and Guided Grad-CAM to generate explanation maps (Figure 1). Grad-CAM (23) produced heatmap that highlighted the important regions in the input image for predicting the hyphae. In this study, the last convolutional layers which offered the best trade-off between high-level semantics and spatial information of the input images were used to compute the weights. Let *y*^*p*^ be the gradient of the score for class “hyphae-positive”, and *A*^*k*^ the feature map k of the last convolutional layer. The gradient of *y*^*p*^ with respect to *A*^*k*^ was computed and averaged by performing global-average-pooled over the total number of elements (indexed by width *i* and height *j*) to produce a weight $\alpha_{k}^{p}$, as shown in Formula (1). The weight $\alpha_{k}^{p}$ represents the importance of the feature map *k* for the positive class.


(1) $$\begin{array}{r} \alpha_{p}^{k}=\frac{1}{Z}\underset{i,j}{\sum }\frac{\partial y^{p}}{\partial A_{ij}^{k}} \end{array}$$

Next, a weighted combination was performed to sum the feature maps. Consequently, the Grad-CAM heatmap was generated by applying the ReLU (rectified linear unit) function to only highlight pixel regions that positively contribute to the positive decision. The formulae are described below as Formula (2).


(2) $$\begin{array}{r} L_{Grad-CAM}^{p}=ReLU\left(\underset{k}{\sum }\alpha_{k}^{p}A^{k}\right) \end{array}$$

**Figure 2:**
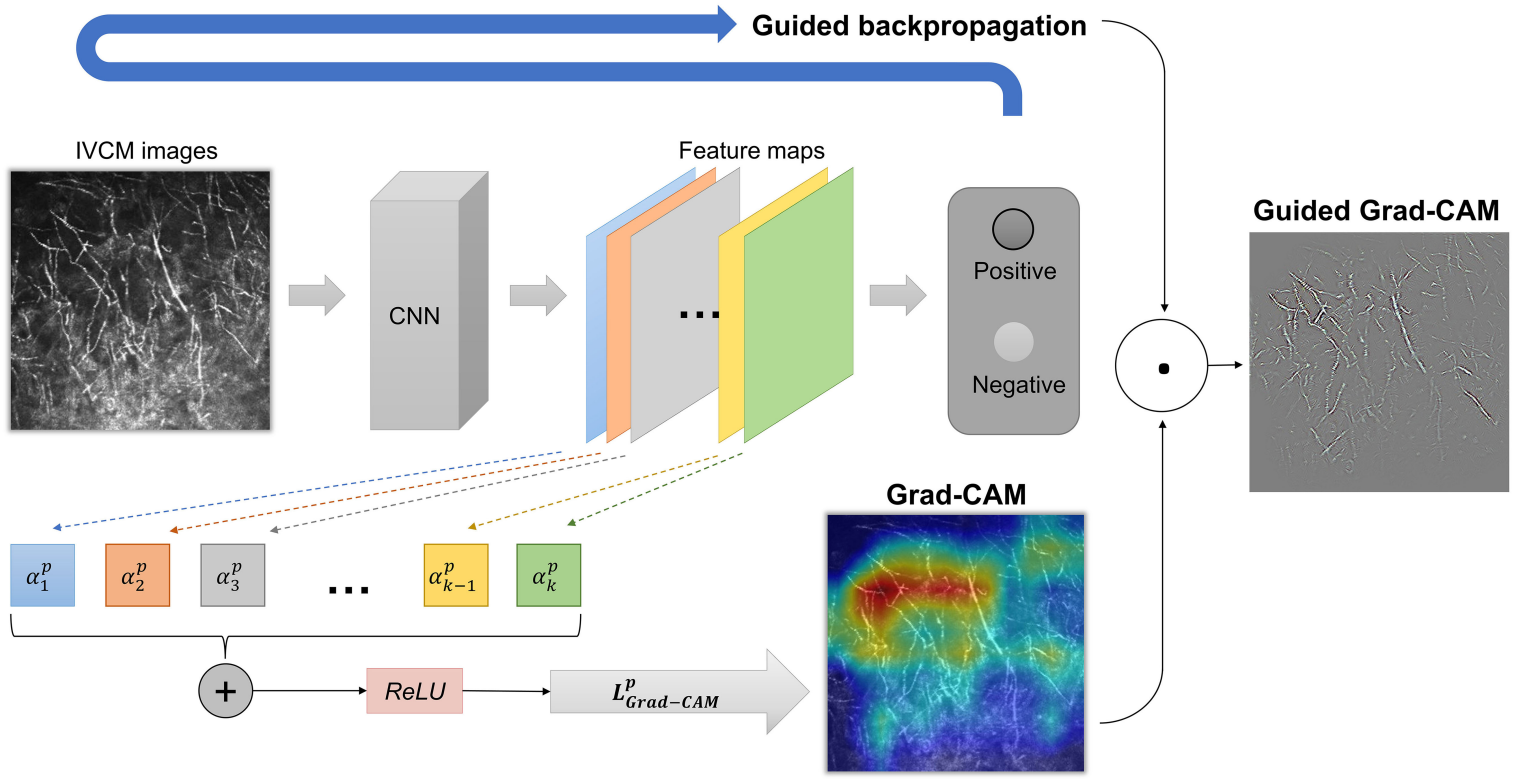
The network architecture of Grad-CAM and Guided Grad-CAM. The weight $\alpha_{k}^{p}$ represents the importance of the feature map *k* for the positive class. A weighted combination and ReLU (rectified linear unit) function were performed to generate Grad-CAM. Finally, element-wise multiplication was performed to fuse Grad-CAM and Guided Backpropagation, thus generating high-resolution Guided Grad-CAM maps.

Although Grad-CAM localized class-discriminative image regions, no fine-grained pixel-space details were available in the heatmaps. Therefore, we used Guided Grad-CAM to further highlight the stripes on the hyphae, which provided pixel-level explanation and help readers to quickly identify the pathogen. Guided Grad-CAM is a combination of Grad-CAM and Guided Backpropagation techniques (20). Guided Backpropagation visualizes the positive gradients by suppressing the negative gradients using ReLU layers. $L_{Grad-CAM}^{p}$ is unsampled to the input image resolution and element-wise multiplication is performed to fuse Grad-CAM and Guided Backpropagation, thus generating high-resolution Guided Grad-CAM maps. The network architecture is shown in Figure 2.

### Ophthalmologist Evaluation

All IVCM images were assessed by nine ophthalmologist readers of varying expertise as follows. The expert ophthalmologist group consisted of three professors with over 10 years of experience in diagnosing corneal diseases. The competent ophthalmologist group comprised three senior ophthalmologists who had over five years of experience in ophthalmology department. The novice ophthalmologist group was composed of three junior ophthalmologists who were in the third year of standardized training for residents of ophthalmology and had been formally trained in IVCM analysis.

Each image was assessed by each reader exactly once, in one of the three conditions: unassisted, AI-assisted, and XAI-assisted. For each reader, the images were equally assigned to each condition so that the same number of images were reviewed for each condition. The assignment of image to reading condition was counter-balanced across groups to make sure that each image was randomly read by one reader in each group in the same condition, thus the reading distributed evenly across reader groups and reading conditions.

**Figure 3:**
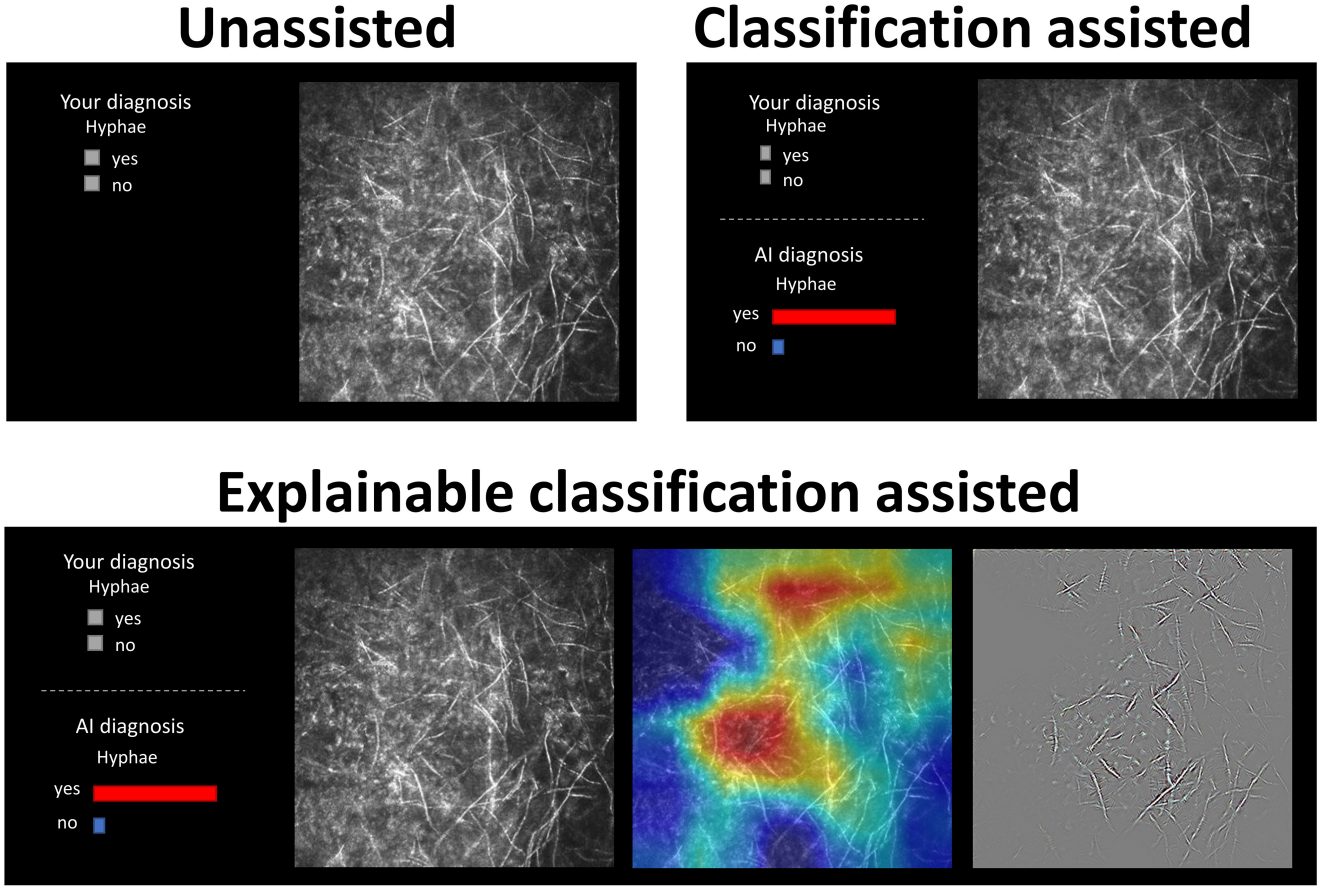
A screenshot of unassisted, AI-assisted, and XAI-assisted conditions. The AI classification results were displayed for both AI-assisted and XAI-assisted conditions, in the form of histograms showing the model prediction probabilities of positive and negative classes. The XAI-assisted conditions included explanatory Grad-CAM heatmap and Guided Grad-CAM maps side by side, in addition to the classification histogram.

The images were displayed in a random order. The AI classification results were displayed for both AI-assisted and XAI-assisted conditions, in the form of histograms showing the model prediction probabilities of positive and negative classes. The XAI-assisted conditions included explanatory Grad-CAM and Guided Grad-CAM maps side by side, in addition to the classification histogram. A screenshot of each condition is shown in Figure 3. The participants were first presented with the original images, and then they had to click on the “AI diagnosis” region to access the classification histograms and explanatory maps.

Readers were masked to the etiology confirmation and reference standard before the reading process. Detailed instructions and guidelines were given to the readers prior to the reading trail. Readers were asked to make a judgment for each image (“Are there fungal hyphae?”). They were told that classification histograms represented the probability of AI prediction, and that the Grad-CAM and Guided Grad-CAM highlighted class-discriminative regions in the images.

Confusion matrices were recorded and the accuracy, sensitivity, and specificity were calculated accordingly. The time required for diagnosis was measured for each reader, but the readers were not informed that the reading time was being recorded.

### Statistical Analysis

Data were analyzed using SPSS (SPSS Version 11.0, IBM-SPSS Inc., Chicago, IL, USA). The AUC of the DL-based model was calculated and compared with the chance level (AUC = 0.5). The statistical significance *P* < 0.05 was considered statistically significant. Comparisons were made using repeated measures analyses of variance (ANOVAs). The Bonferroni *post-hoc* test was used to correct for multiple comparisons. The significance was set at 0.05/N, where N is the number of tests used.

## Results

### Model Performance

**Figure 4:**
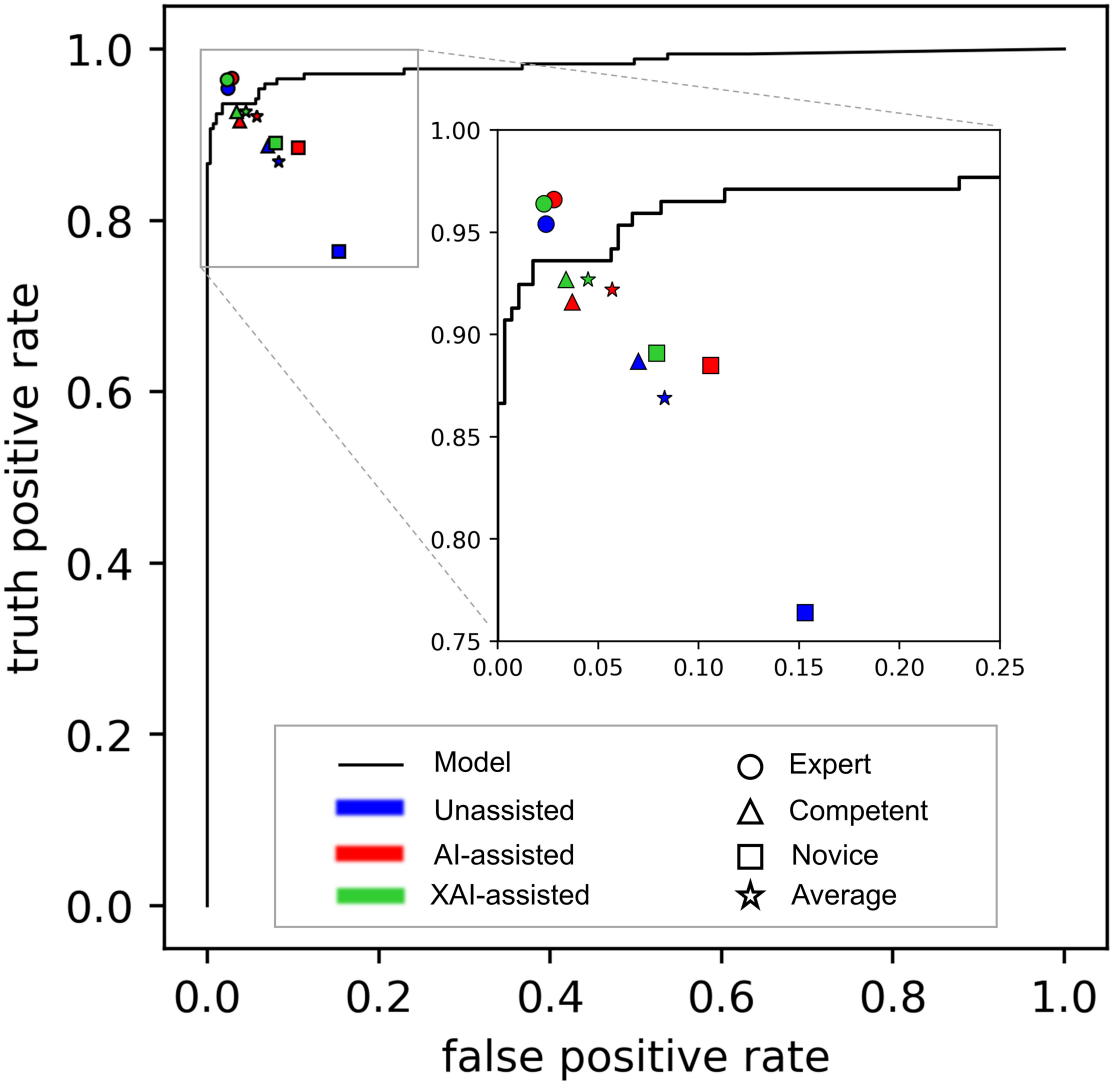
The performance of the model alone and readers in different reading conditions. The receiver-operating characteristic (ROC) curve of the DL-based model is depicted as black line, showing the overall performance of the model alone. The performance of readers is distinguished by different shapes and colors. The shapes represent different groups of reader (Round: expert ophthalmologists; triangle: competent ophthalmologists; square: competent ophthalmologists; pentagram: an average of all ophthalmologists). Filled colors represent different reading conditions (blue: unassisted; red: AI-assisted; green: XAI-assisted).

The receiver-operating characteristic (ROC) curve of the DL-based model is shown in Figure 4. The model achieved an area under the ROC curve (AUC) of 0.983 (*P* < 0.001) and accuracy, sensitivity, and specificity of 0.965, 0.936, and 0.982, respectively. Visually, Grad-CAM and Guided Grad-CAM localized roughly the same image regions where hyphae aggregated. With the gradients flowing back, the Grad-CAM yielded a rough visualization result. On the contrary, through Guided Backpropagation, we made full use of the pixel-level information of the input image and obtained finer visualization.

### Accuracy Evaluation

**Figure 5:**
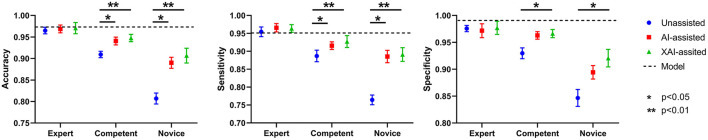
The accuracy, sensitivity, and specificity of model alone and readers in different reading conditions.

The results in accuracy, sensitivity, and specificity are shown in Table 1. The average accuracy for all readers without assistance was 0.894 (95% confidence interval (CI) 0.888–0.899), which was improved to 0.933 (95% CI 0.927–0.939) with AI assistance (*p* < 0.001) and 0.942 (95% CI 0.933–0.951) with XAI assistance (*p* < 0.001). The changes in accuracy across reading conditions are displayed in Figure 5. For the expert ophthalmologists, the accuracy was 0.966 (95% CI 0.955–0.977) in unassisted condition, which was not statistically different when compared with that in AI-assisted (0.969, 95% CI 0.958–0.979) and XAI-assisted conditions (0.971, 95% CI 0.946–0.996). For the competent ophthalmologists, the accuracy improved from 0.910 (95% CI 0.901–0.919) to 0.941 (95% CI 0.925–0.956) with AI assistance (*p* = 0·032) and to 0.948 (95% CI 0.934–0.961) with XAI assistance (*p* = 0.006). For the novice ophthalmologists, the accuracy improved from 0.807 (95% CI 0.783–0.831) to 0.890 (95% CI 0.866–0.914) with AI assistance (*p* = 0.041) and to 0.907 (95% CI 0.869–0.944) with XAI assistance (*p* = 0.009).

**Table 1:** The accuracy, sensitivity and specificity of readers in unassisted, AI-assisted, and XAI-assisted reading conditions^a^.

	**Accuracy**			**Sensitivity**			**Specificity**		
	**Unassisted**	**AI-assisted^b^**	**XAI-assisted^b^**	**Unassisted**	**AI-assisted^b^**	**XAI-assisted^b^**	**Unassisted**	**AI-assisted^b^**	**XAI-assisted^b^**
Expert ophthalmologist	0.966 (0.955–0.977)	0.969 (0.958–0.979) *p =* 0.999	0.971 (0.946–0.996) *p =* 0.650	0.954 (0.926–0.983)	0.966 (0.941–0.990) *p =* 0.999	0.964 (0.942–0.985) *p =* 0.152	0.976 (0.968–0.983)	0.972 (0.944–0.999) *p =* 0.999	0.97 (0.9950–1.004) *p =* 0.999
Competent ophthalmologist	0.910 (0.901–0.919)	**0.941s** **(0.925–0.956)** ***p** **=*** **0.032**	**0.948** **(0.934–0.961)** ***p** **=*** **0.006**	0.887 (0.852–0.922)	**0.916** **(0.894–0.937)** ***p** **=*** **0.036**	**0.927** **(0.891–0.964)** ***p** **<*** **0.001**	0.930 (0.910–0.950)	0.963 (0.951–0.975) *p =* 0.142	**0.966 (0.959–0.974)** ***p** **=*** **0.022**
Novice ophthalmologist	0.807 (0.783–0.831)	**0.890** **(0.866–0.914)** ***p** **=*** **0.041**	**0.907** **(0.869–0.944)** ***p** **=*** **0.009**	0.764 (0.736–0.793)	**0.885** **(0.847–0.923)** ***p** **=*** **0.007**	**0.891** **(0.849–0.933)** ***p** **=*** **0.002**	0.847 (0.812–0.881)	0.894 (0.868–0.920) *p =* 0.225	**0.921** **(0.885–0.956)** ***p** **=*** **0.035**
Average	0.894 (0.888–0.899)	**0.933** **(0.927–0.939)** ***p** **<*** **0.001**	**0.942** **(0.933–0.951)** ***p** **<*** **0.001**	0.869 (0.858–0.879)	**0.922** **(0.913–0.932)** ***p** **<*** **0.001**	**0.927** **(0.916–0.939)** ***p** **<*** **0.001**	0.917 (0.910–0.925)	**0.943** **(0.935–0.951)** ***p** **=*** **0.014**	**0.955** **(0.946–0.963)** ***p** **<*** **0.001**

*AI, artificial intelligence; XAI, explainable artificial intelligence. Statistically significant values are shown in bold*.

a *The results are expressed as the mean (95% Confidence Interval)*.

b *The statistical significance level of the index in AI/XAI-assisted conditions compared to that in unassisted condition is shown as p*.

An overview of sensitivity (True positive rate) and specificity (1-False positive rate) is shown in Figure 4. In general, performance in XAI-assisted condition was better than that in AI-assisted condition, and both better than that without assistance. The influence of reading conditions was more prominent on sensitivity than on specificity (shown in Figure 5). For the expert ophthalmologist group, there was no statistical difference in sensitivity among the reading conditions. For the competent and novice group, the sensitivity for both forms of assistance exceeded that of unassisted reads (competent ophthalmologist 0.887, 95% CI 0.852–0.922; novice ophthalmologist 0.764, 95% CI 0.736–0.793), with the XAI-assisted sensitivity (competent ophthalmologist 0.927, 95% CI 0.891–0.964, *p* <0.001; novice ophthalmologist 0.891, 95% CI 0.849–0.933, *p* = 0.002) being slightly higher than AI-assisted sensitivity (competent ophthalmologist 0.916, 95% CI 0.894–0.937, *p* = 0.036; novice ophthalmologist 0.885, 95% CI 0.847–0.923, *p* = 0.007). The improvement of sensitivity was more evident in the novice ophthalmologist group than in the competent ophthalmologist group. The specificity with XAI assistance was significantly higher than that without assistance in the competent (unassisted 0.930, 95% CI 910–0.950; XAI-assisted 0.966, 95% CI 0.959–0.974; *p* = 0.022) and novice ophthalmologist groups (unassisted 0.847, 95% CI 0.812–0.881; XAI-assisted 0.921, 95% CI 0.885–0.956; *p* = 0.035), while no statistically significant difference existed between AI-assisted and unassisted conditions.

### Efficiency Evaluation

**Figure 6:**
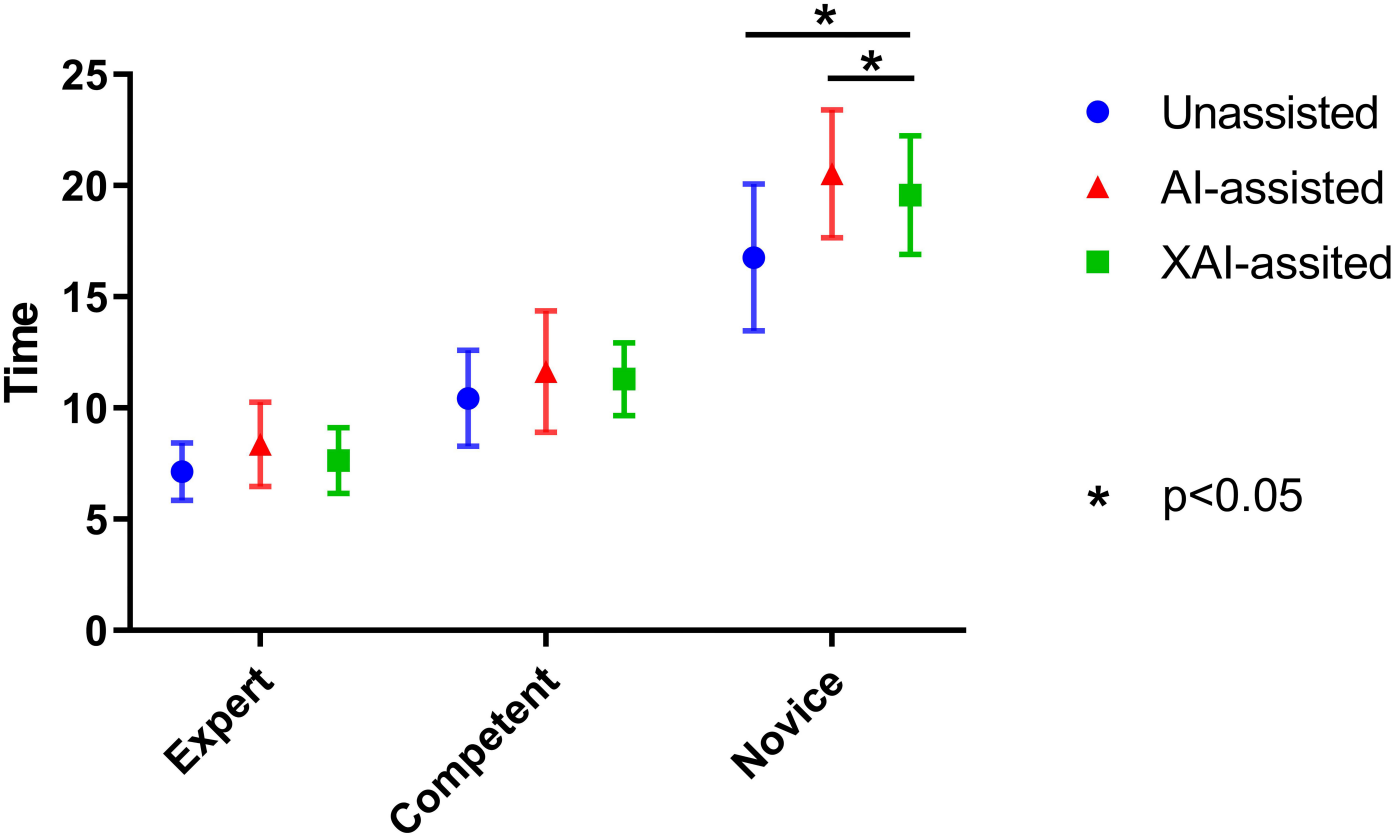
The average time that readers spent on each image in different reading conditions.

On average, novice ophthalmologists spent more time per image with AI assistance than without assistance (*P* = 0.040). For novice ophthalmologists, the time spent with XAI assistance was significantly less than with AI assistance (*P* = 0.045). Although the time spent with XAI assistance tended to be higher than that without assistance, the difference was not statistically significant (*P* = 0.092). The same trends were observed for competent and expert ophthalmologists but the differences were statistically insignificant (shown in Figure 6).

## Discussion

This study evaluated the impact of visually explainable AI on IVCM image analysis. The results showed that AI and XAI helped improve reading accuracy, and the effect was more pronounced for inexperienced ophthalmologists compared to experienced ophthalmologists. The assistance of AI increased sensitivity, but not at the expense of specificity. The addition of explanatory maps further amplified the positive effect. Although AI assistance prolonged the average time per image, the application of explanatory maps reduced the prolongation.

We noted that reading without assistance was generally high in specificity but low in sensitivity, which implied that readers might tend to judge an image as “negative”, rather than “positive”, in cases of ambiguous images. The missed diagnosis might happen easily under the situations. The assistance of AI reduced the number of false negative samples by helping readers correctly recognize the true positives, without increasing corresponding false-positive errors. The possible reason is that a positive model prediction might arise the attention of readers to identify occult lesions that otherwise would be easily overlooked.

Explanatory maps contributed to improving both sensitivity and specificity. For the true positive samples that were correctly predicted by the model (TP), the explanatory maps highlighted the morphology and location of fungal hyphae, providing an interpretable and explainable basis of AI decision-making, thus improving the user's trust in the model's prediction. For the true negative samples that were incorrectly predicted by the model (FP), the explanatory maps displayed meaningless spatial information, which helped the user to identify the model misdiagnosis and therefore avoided the possible interference caused by model errors.

In the present study, Grad-CAM and Guided Grad-CAM help ophthalmologists determine whether the model results are credible by highlighting the important regions that lead to model decision. Recent studies have proposed uncertainty measurement as an efficient method for the evaluation of model confidence (26). Uncertainty measurement provides an estimation of pixel-wise uncertainties for image segmentation results, which enables an easy decision to accept or reject the model outcome based on a certain uncertainty level. This provides new ideas for the research of XAI and we are going to explore this approach in IVCM image analysis in our future research.

The impact of AI and XAI on the accuracy was noted to vary according to the degree of clinical experience. The inexperienced readers may be more likely to profit from the XAI system. With the assistance of XAI, the accuracy of novice ophthalmologist was increased to approximately competent level, and the accuracy of competent ophthalmologists reached close to that of expert. Although IVCM is greatly helpful for the diagnosis of corneal diseases, it is far from universal in many locations. One of the reasons is the scarcity of reading ophthalmologists. The explainable system is appropriate for teaching IVCM analysis skills. The validated model can provide timely monitoring and feedback to inexperienced readers, and the explanatory maps can help quickly identify the important features as a basis for judgment, thus help reduce study time and corresponding costs.

While AI assistance prolonged the mean reading time per image, it was within acceptable ranges considering the contribution of AI to the accuracy improvement. This can be easily understood because the classification histograms and explanatory maps were shown after the original images, and it took more time to reflect on divergent results. The addition of explanatory maps visualized the AI diagnostic basis, thus reduced the hesitation time. It should be mentioned that the readers were not instructed to complete the reading task as fast as possible, and were not informed that the reading time would be used as an auxiliary evaluation index, which might influence the results. Interestingly, although mean time per image increased, AI still has high potential for improving efficiency in the clinical setting given that AI may help to rapidly screen out hyphae-positive images from hundreds of images consecutively collected from each eye. The effect of AI on reading efficiency deserves a further study.

The proposed algorithm framework is general, which can be extended to other pathogens such as Acanthamoeba and Candida. IVCM images show Acanthamoeba cysts and trophozoites in Acanthamoeba keratitis (27), and show spore and pseudohyphae in Candida keratitis (28). A deep learning model can learn either these features or novel features to predict Acanthamoeba and Candida keratitis.

There were several limitations existed in this study. First, this is a single-center study performed in a limited number of readers, selection bias was inevitable. A multicenter study with more participants is needed in the future to generate more robust results. Second, we included a balanced number of positives vs. negatives with a ratio of approximately 1:1, thus the percentage of positivity in this study differed from that in a real-world setting. The diverse prevalence might significantly a?ect the sensitivity and specificity between datasets, thereby reducing the generalizability of our findings. Third, IVCM images were presented in random order in this study, but instead in order of scanning in actual clinical settings. Randomization of order excluded contextual information of adjacent images and so the performance of ophthalmologists could be underestimated. Therefore, additional studies are required to validate the role of AI in the analysis of image sequences. Finally, this study failed to compare the sensitivity between microbiological tests and IVCM. In this study, the microbiological tests were used as the gold standard. All positive images were collected from eye with positive smear/culture of filamentous fungi and were adjudicated by corneal specialists that hyphal structures were present in the observation field. All negative images were collected from eye with negative smear/culture of filamentous fungi and were ensured that no hyphae were included in the images. Therefore, cases with negative fungal smear/culture and positive hyphae fundings in IVCM were not included in the study, and the question of whether patients with negative microbiological tests could benefit from IVCM with DL was not addressed in this study. Despite this, the study provides an important framework for the future researches. Further studies will incorporate hyphae-positive images with negative microbiological results to assess the evaluation of AI-assisted IVCM as a means to complement microbiological tests.

## Conclusion

AI has shown great promise in improving the accuracy of ophthalmologists in terms of FK detection using IVCM images. The inexperienced readers are more likely to benefit from the XAI system. With better interpretability and explainability, XAI-assistance can boost ophthalmologist performance beyond what is achievable by the reader alone or with black-box AI assistance. The present study extends our understanding of the role of AI in medical image analysis.

## Data Availability Statement

The original contributions presented in the study are included in the article/supplementary material, further inquiries can be directed to the corresponding author/s.

## Ethics Statement

This study was approved by the Ethics Committee of The People's Hospital of Guangxi Zhuang Autonomous Region, China. The approval number is KY-SY-2020-1. Informed consent was waived because of the anonymized usage of images. No potentially identifiable human images or data is presented in this study.

## Author Contributions

FX conceived the research and wrote the manuscript. LJ, WH, and GH performed the analyses. YQ, RL, and XP contributed to algorithm optimization. YH, FT, JL, and YL contributed to data collection and measurements. SZ and ML were involved quality management. QC and NT provided overall supervision, edited the manuscript, and undertook the responsibility of submitting the manuscript for publication. All authors read and approved the final manuscript.

## Funding

This study was supported by Guangxi Promotion of Appropriate Health Technologies Project (No. S2019084) and Guangxi Clinical Ophthalmic research center (No. GuikeAD19245193).

## Conflict of Interest

YQ was employed by company China-ASEAN Information Harbor Co., Ltd. The remaining authors declare that the research was conducted in the absence of any commercial or financial relationships that could be construed as a potential conflict of interest.

## Publisher's Note

All claims expressed in this article are solely those of the authors and do not necessarily represent those of their affiliated organizations, or those of the publisher, the editors and the reviewers. Any product that may be evaluated in this article, or claim that may be made by its manufacturer, is not guaranteed or endorsed by the publisher.

## References

[1] Stapleton F. The epidemiology of infectious keratitis. Ocular Surface. (2021). 21:00089–6. 10.1016/j.jtos.2021.08.007 34419639

[2] Whitcher JP Srinivasan M Upadhyay MP. Corneal blindness: a global perspective. Bull World Health Organ. (2001) 79:214–21. 11285665 PMC2566379

[3] Green MD Apel AJ Naduvilath T Stapleton FJ. Clinical outcomes of keratitis. Clin Experiment Ophthalmol. (2007) 35:421–6. 10.1111/j.1442-9071.2007.01511.x 17651246

[4] Leal SM Rodino KG Fowler WC Gilligan PH. Practical guidance for clinical microbiology laboratories: diagnosis of ocular infections. Clin Microbiol Rev. (2021) 34:e0007019. 10.1128/CMR.00070-19 34076493 PMC8262805

[5] Wang YE Tepelus TC Vickers LA Baghdasaryan E Gui W Huang P. Role of *in vivo* confocal microscopy in the diagnosis of infectious keratitis. Int Ophthalmol. (2019) 39:2865–74. 10.1007/s10792-019-01134-4 31209694

[6] Labbé A Khammari C Dupas B Gabison E Brasnu E Labetoulle M. Contribution of *in vivo* confocal microscopy to the diagnosis and management of infectious keratitis. Ocul Surf. (2009) 7:41–52. 10.1016/S1542-0124(12)70291-4 19214351

[7] Chidambaram JD Prajna NV Palepu S Lanjewar S Shah M Elakkiya S. Cellular morphological changes detected by laser scanning *in vivo* confocal microscopy associated with clinical outcome in fungal keratitis. Sci Rep. (2019) 9:8334. 10.1038/s41598-019-44833-9 31171825 PMC6554396

[8] Das S Samant M Garg P Vaddavalli PK Vemuganti GK. Role of confocal microscopy in deep fungal keratitis. Cornea. (2009) 28:11–3. 10.1097/ICO.0b013e318181cff7 19092397

[9] Kanavi MR Javadi M Yazdani S Mirdehghanm S. Sensitivity and specificity of confocal scan in the diagnosis of infectious keratitis. Cornea. (2007) 26:782–6. 10.1097/ICO.0b013e318064582d 17667609

[10] Takezawa Y Shiraishi A Noda E Hara Y Yamaguchi M Uno T. Effectiveness of *in vivo* confocal microscopy in detecting filamentous fungi during clinical course of fungal keratitis. Cornea. (2010) 29:1346–52. 10.1097/ICO.0b013e3181cd3c84 20847667

[11] Hau SC Dart JK Vesaluoma M Parmar DN Claerhout I Bibi K. Diagnostic accuracy of microbial keratitis with in vivo scanning laser confocal microscopy. Br J Ophthalmol. (2010) 94:982–7. 10.1136/bjo.2009.175083 20538659

[12] Lv J Zhang K Chen Q Chen Q Huang W Cui L. Deep learning-based automated diagnosis of fungal keratitis with in vivo confocal microscopy images. Ann Transl Med. (2020) 8:706. 10.21037/atm.2020.03.134 32617326 PMC7327373

[13] Xu F Qin Y He W Huang G Lv J Xie X. A deep transfer learning framework for the automated assessment of corneal inflammation on *in vivo* confocal microscopy images. PLoS ONE. (2021) 16:e0252653. 10.1371/journal.pone.0252653 34081736 PMC8174724

[14] Tjoa E Guan C. A survey on explainable artificial intelligence (XAI): toward medical XAI. IEEE Transac Neural Netw Learn Syst. (2020) 32:4793–4813. 10.1109/TNNLS.2020.3027314 33079674

[15] Zeiler MD Fergus R. Visualizing and understanding convolutional networks. In: European Conference on Computer Vision, Springer, Cham. (2014). p. 818–33. 10.1007/978-3-319-10590-1_53

[16] Goodfellow IJ Shlens J Szegedy C. Explaining and harnessing adversarial examples. arXiv preprint arXiv:1412.6572. (2014).

[17] Simonyan K Vedaldi A Zisserman A. Deep inside convolutional networks: visualising image classification models and saliency maps. arXiv preprint arXiv:1312.6034. (2013).

[18] Springenberg JT Dosovitskiy A Brox T Riedmiller M. Striving for simplicity: the all convolutional net. arXiv preprint arXiv:1412.6806. (2014).

[19] Bach S Binder A Montavon G Klauschen F Müller KR Samek W. On pixel-wise explanations for non-linear classifier decisions by layer-wise relevance propagation. PLoS ONE. (2015) 10:e0130140. 10.1371/journal.pone.0130140 26161953 PMC4498753

[20] Selvaraju RR Cogswell M Das A Vedantam R Parikh D Batra D. Grad-cam: Visual explanations from deep networks via gradient-based localization. In: Proceedings of the IEEE International Conference on Computer Vision. (2017). p. 618–26. 10.1109/ICCV.2017.7427295638

[21] Chen H Lundberg S Lee SI. Explaining models by propagating Shapley values of local components. In: Explainable AI in Healthcare and Medicine Springer, Cham. (2021). p. 261–70. 10.1007/978-3-030-53352-6_24

[22] Yang G Ye Q Xia J. Unbox the black-box for the medical explainable ai via multi-modal and multi-centre data fusion: a mini-review, two showcases and beyond. arXiv preprint arXiv:2102.01998. (2021). 10.1016/j.inffus.2021.07.016 PMC8459787 34980946

[23] Zhou B Khosla A Lapedriza A Oliva A Torralba A. Learning deep features for discriminative localization. In: Proceedings of the IEEE Conference on Computer Vision and Pattern Recognition. (2016). p. 2921–9. 10.1109/CVPR.2016.31927295638

[24] Montavon G Lapuschkin S Binder A Samek W Müller KR. Explaining nonlinear classification decisions with deep taylor decomposition. Pattern Recognit. (2017) 65:211–22. 10.1016/j.patcog.2016.11.008

[25] Ye Q Xia J Yang G. Explainable AI for COVID-19 CT classifiers: an initial comparison study. arXiv preprint arXiv:2104.14506. (2021). 10.1109/CBMS52027.2021.0010327295638

[26] Liu Y Yang G Hosseiny M Azadikhah A Mirak SA Miao Q. Exploring uncertainty measures in Bayesian deep attentive neural networks for prostate zonal segmentation. IEEE Access. (2020) 8:151817–28. 10.1109/ACCESS.2020.3017168 33564563 PMC7869831

[27] Wei Z Cao K Wang L Baudouin C Labbé A Liang Q. Corneal changes in acanthamoeba keratitis at various levels of severity: an *in vivo* confocal microscopic study. Transl Vision Sci Technol. (2021) 10:10. 10.1167/tvst.10.7.10 34110388 PMC8196423

[28] Le Q Wu D Li Y Ji J Cai R Xu J. Early-onset candida glabrata interface keratitis after deep anterior lamellar keratoplasty. Optom Vision Sci. 92:e93–6. 10.1097/OPX.0000000000000565 25822017

